# Marginal Bone Level Changes in Full‐Arch Rehabilitation: Digital Versus Analog Protocols—A 5‐Year Retrospective Study

**DOI:** 10.1111/cid.70080

**Published:** 2025-07-28

**Authors:** Nicola De Angelis, Paolo Pesce, Vito Carlo Alberto Caponio, Giulia Santamaria, Oriana Spanu, Maria Menini

**Affiliations:** ^1^ Department of Surgical Sciences and Integrated Diagnostics University of Genova Genova Italy; ^2^ Department of Clinical and Experimental Medicine University of Foggia Foggia Italy

**Keywords:** dental implants, fixed, immediate dental implant loading, impression techniques, intraoral scanners, marginal bone loss, prosthodontics

## Abstract

**Introduction:**

This retrospective study compares the clinical outcomes of analog impressions versus intraoral scanning in full‐arch immediate loading rehabilitations. Specifically, it evaluates peri‐implant marginal bone level (MBL) changes at different time intervals (implant placement, loading, and at 2 and 5 years), as well as rates of mechanical and prosthetic complications.

**Materials and Methods:**

The study included 62 patients who underwent full‐arch rehabilitation with immediate implant placement between 2019 and 2020. Patients were divided into two groups: analog impression and digital intraoral scanning. All patients were rehabilitated with fixed titanium‐PMMA screw retained restorations. Bone level was assessed through standardized intraoral radiographs at key time points. Additional parameters recorded included procedural time, prosthetic complications, and implant failures. Statistical analyses involved repeated measures ANOVA and post hoc Bonferroni tests.

**Results:**

The follow‐up period was 5 years. Implant survival was 99.6%. No significant differences were found in prosthetic complications. MBL was slightly higher in the analog group at baseline (mean = 0.21, SD = 0.04 vs. digital mean = 0.17, SD = 0.04, *t*‐test *p*‐value < 0.001) than in the digital group. Despite this, the overall bone loss remained within clinically acceptable limits during the follow‐up period. Digital impressions significantly reduced procedural time compared to analog methods.

**Conclusions:**

Both impression techniques provided satisfactory clinical outcomes. Digital impressions demonstrated efficiency advantages but were associated with slightly greater bone loss over time. Analog impressions remain a reliable standard for full‐arch immediate loading rehabilitations, though digital methods show promise for improved patient experience. Further randomized, long‐term studies are needed.

**Clinical Significance:**

Digital impressions offer a faster and more comfortable workflow for full‐arch immediate loading rehabilitations, potentially improving patient compliance. However, their association with slightly greater bone loss warrants further investigation to optimize long‐term stability.

## 1 Introduction

The spread of full‐arch immediate loading rehabilitations has revolutionized the field of dentistry, offering patients the possibility to achieve fixed restorations and fully restore masticatory function in a quick and lasting manner. Studies have shown that even a reduced number of implants can be sufficient, provided that they are adequately long and that loads, particularly for non‐axial implants, are correctly distributed [1, 2].

This procedure is based on the principle of mechanical bio‐inertia, which optimizes implant stability and promotes osseointegration. In this context, it has been observed that micromovements at the bone‐implant interface, when kept within a physiological range, not only do not compromise osseointegration [3], but also contribute to qualitative improvements in peri‐implant bone, with a significant reduction in marginal bone loss [2].

Immediate loading is highly valued by patients, as it allows them to maintain function and esthetics throughout the treatment, enabling the continuation of daily activities and reducing social, occupational, and psychological impact. This approach spares patients from the use of removable temporary prostheses and reduces the number of specialist visits [4, 5, 6].

In the same session in which the implants are placed, the prosthetic restorations are fabricated using various traditional impression techniques, which have been sufficiently validated in the literature and demonstrate reliability and accuracy [7, 8].

The pick‐up impression technique is the most well‐known and widely used, typically performed with plaster material in case of full‐arch rehabilitations. Plaster, being a rigid material, has shown ideal results in terms of precision and stability compared to other impression materials [8]. However, it presents some disadvantages, including limited adaptability to adjustments during the setting phase in case of undercuts and in case of residual teeth, accompanied by discomfort for the patient. These limitations can lead to a limited use of this technique and are mostly employed by expert prosthodontists.

Recently, the introduction of intraoral scanners has seen rapid expansion, especially for taking impressions of single units and/or three‐ to four‐unit implant‐supported bridges [9, 10, 11]. However, the accuracy of this innovative impression technique has not yet been fully validated for full‐arch rehabilitations [12]. In a retrospective study published in 2023, the two techniques (analog and digital impressions) were compared. Higher patients' acceptance was recorded for the digital protocol, without differences in the recorded precision of the prosthetic structures [13]. An alternative approach is to use fully digital workflows, which eliminate the need for postoperative impressions while allowing the delivery of a prefabricated temporary bridge. In such cases, however, guided surgery is necessary to ensure the predictability of implant positioning, allowing for an optimal fit of the prosthesis [14].

The accuracy of impression techniques aims to reduce the frequency of technical complications at the time of delivery of the prosthesis or during the follow‐up [15, 16, 17]. This is important because, although the implant survival rate in full‐arch rehabilitations is satisfactory, technical complications may arise, such as screw loosening and chipping of the superstructure, which are quite common [18, 19, 20].

Marginal bone level (MBL) is considered one of the main outcomes to take into account while assessing implant success, both in partial and full‐arch rehabilitations [21, 22, 23, 24].

MBL has been thoroughly investigated by lots of clinical studies, and several systematic reviews evaluated its variation connected to implant position, implant surface as well as the type of abutment connection, history of periodontitis, smoking habits, and other factors [14, 15]. To the authors knowledge, while MBL might be affected by the above‐mentioned variables, no direct interactions have been found between peri‐implant MBL changes and impression technique.

In particular, in full‐arch immediate loading rehabilitations, peri‐implant bone loss might be particularly high during the first year after implant insertion, due to bone remodeling after the surgical trauma and immediate loading of implants that are not osseointegrated yet. Usually, a steady state condition is observed after the first year [1]. A clinical comparative study found less bone loss in full‐arch immediate loading rehabilitations compared to traditional delayed loading protocols, probably due to the mucosal load of the provisional removable prosthesis in the delayed loading group [25].

In addition, in a systematic review published in 2012 [26], the authors investigated bone loss in tilted versus upright implants used for immediate full‐arch rehabilitations without reporting statistically significant differences.

Since full‐arch immediate loading rehabilitation is often supported by a reduced number of implants (typically 4–6), bone loss and subsequent implant failure might compromise the entire rehabilitation. It is therefore particularly important to reduce bone loss as much as possible.

A preliminary study [13] analyzed the fit of immediate full‐arch prostheses, fabricated using conventional or digital impressions. A statistically significant difference in prosthesis fit was found between digital and conventional impression groups, as well as the MBL was higher for patients where the digital impression was taken.

Although a direct correlation between prosthesis misfit and bone loss has not been demonstrated yet, impression technique might affect bone loss in full‐arch immediate loading rehabilitations, since impression accuracy is one of the main factors influencing the passive fitting of the prosthesis. In addition, prosthesis misfit might be correlated with increased technical complications. However, clinical studies investigating the correlation between impression technique and peri‐implant bone loss and technical complications are scarce.

In this scenario, the aim of the present retrospective study is to compare peri‐implant MBL at different time intervals including implant positioning (T0), implant loading (T1) and at 2 years (T3) and 5 years (T4) of follow‐up. Rates of mechanical and prosthetic complications (such as fracture of the structures and/or of the veneering material) were also recorded.

## 2 Materials and Methods

This retrospective study was conducted in full compliance with the Declaration of Helsinki and was approved by the Ethical Committee of the University of Genoa (CERA 2024/83). The study was reported following STROBE Guidelines for clinical studies (Data S1). Records were retrieved from the private office where the treatments were done, including informed consent, covering the study's purpose, procedure, risks, and benefits, and additional signed releases, which were collected for the use of patient images and radiographies in the study.

Patients underwent full‐arch rehabilitation with simultaneous extractions and immediate dental implant placement [1, 27, 28] without restrictions of position and site (upper and lower jaw) in a private practice in Acqui Terme (AL, Italy) between January 2019 and January 2020 and were followed for the consecutive 5 years. To be included in the analysis, patients had to meet the following criteria: treated with full‐arch immediate loading rehabilitation for at least 5 years; jaws had to be at least 12 mm high in the anterior region and 4 mm wide, in order to allow the placement of four implants (two straight and two tilted); no general contraindications to implant surgery (i.e., immunosuppressed or immunocompromised patients, history of irradiation in the head or neck area, uncontrolled diabetes, pregnant or lactating women); in contrast, the following patients were excluded: patients with untreated periodontitis and poor oral hygiene and motivation; substance abuse; heavy smokers (more than 10 cigarettes/day); psychiatric disorders or unrealistic expectations; acute infection in the site intended for implant placement; under treatment or previous treatment with intravenous amino‐bisphosphonates; participation in other clinical trials interfering with the present protocol; sites, judged by the investigator, with a bone volume insufficient to guarantee at least 1.5 mm of bone all around the implant.

### 2.1 Sample Size Calculation

The sample size was estimated based on a previous study [15] comparing bone loss at 6, 12, and 24 months in patients undergoing full‐arch screw‐retained maxillary rehabilitation based on a digital versus analog protocol (respectively, mean bone loss and standard deviation for digital vs. analog impression at 6, 12, and 24 months = 1.03 ± 0.32 vs. 0.99 ± 0.48; 1.04 ± 0.56 vs. 1.08 ± 0.52; and 1.07 ± 0.66 vs. 1.11 ± 0.54). As far as we know, no study reported observations at 5 years follow‐up comparing digital versus analog impressions. G*Power 3.1.9.7 was used for estimating a priori sample size. ANOVA, repeated measures, between factors from *F* tests family was picked, and computation was estimated based on a large effect size (0.4), as previously stated [16, 17], and α error probability was set to 0.05 and power *β* to 0.80. As our study included two groups and four timepoints of measurements, these values were input into G*Power, together with the correlation value from the repeated measures coming from the above‐mentioned study (0.81). Total sample size resulted in 46, 23 each group.

### 2.2 Surgical Procedure

All surgical and prosthodontic procedures were carried out by the same clinician with more than 5 years of experience in full‐arch immediate loading rehabilitation (N.D.A.). The surgeries were conducted under local anesthesia using Articaine (40 mg/mL) combined with Epinephrine (1:100 000).

**FIGURE 1:**
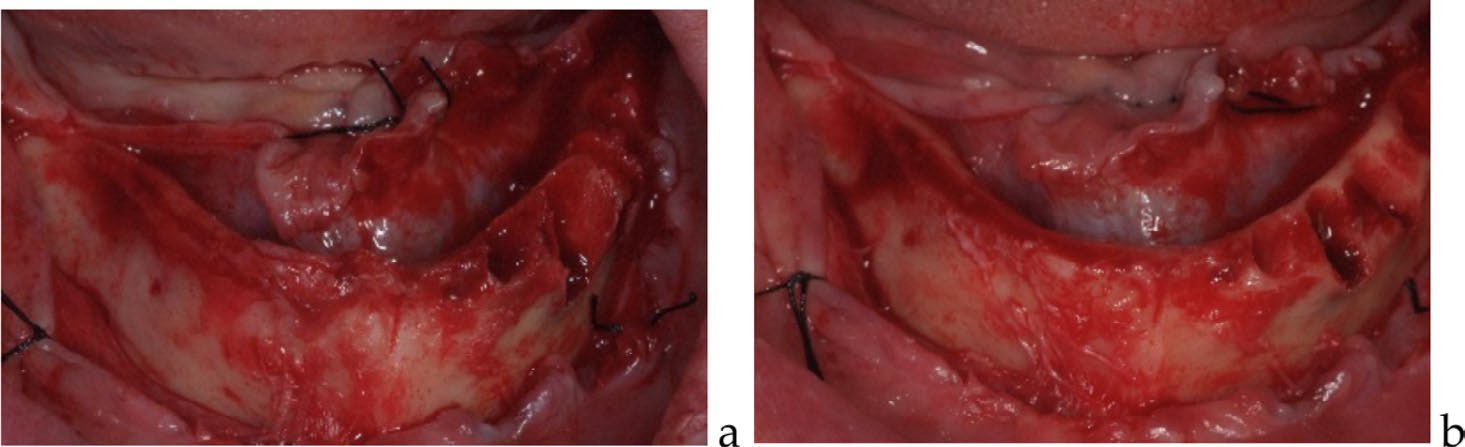
(a) Intraoral pictures of a mandibular case after teeth extraction and flap elevation; (b) a minimal ostectomy was performed in order to compensate for bone discrepancies.

A full‐thickness flap was elevated from molar to molar without surgical guide, extending to the buccal aspects of both the maxilla and mandible while carefully isolating and preserving anatomical structures. When necessary, minimal ostectomy was performed to address bone irregularities (Figure 1).

Following flap elevation, the clinician prepared the implant sites using a sequence of drills as recommended by the manufacturer (Straumann Holding AG, Basel, Switzerland—BLT SLActive surface). One or two teeth that did not interfere with the implant placement were left to facilitate the matching of preoperative and postoperative impressions and extracted immediately after.

**FIGURE 2:**
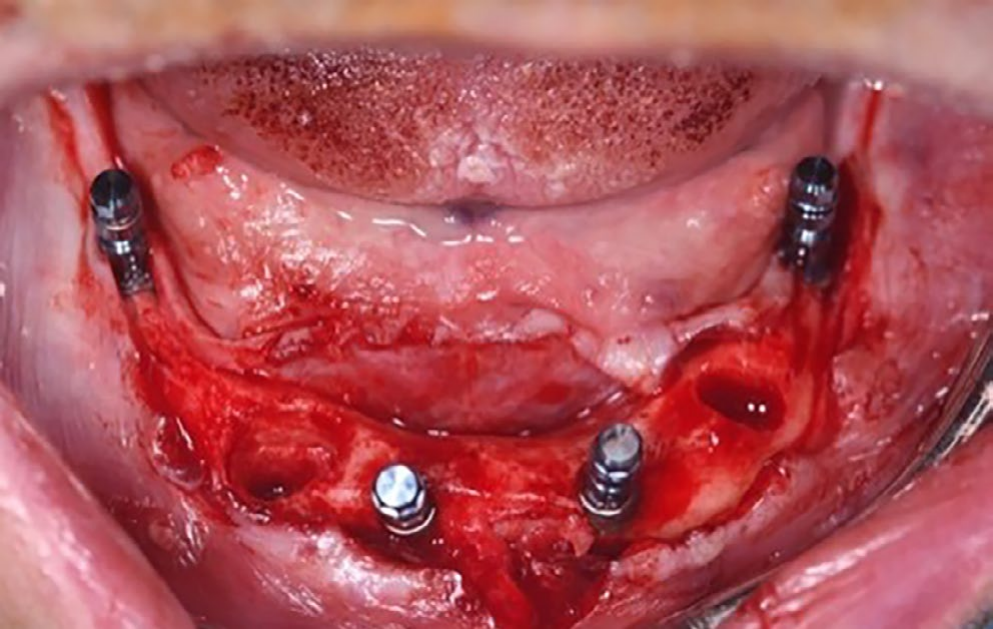
Intraoral picture of implant placement.

When the bone was judged soft by the clinician, the implant sites were undersized when necessary to enhance primary stability, achieved by bone drilling while omitting the final dedicated drill for soft bone class 4. Surgical implant placement was carried out following the Columbus Bridge Protocol, as outlined by Tealdo et al. in 2011 [29]. The two distal implants were mesio‐distally tilted in the case of atrophic jaws or placed upright in the case of sufficient bone volume and had a minimum length of 10 mm (Figure 2).

Following implant placement, multi‐unit abutments were attached to the implants at 25 Ncm, following this configuration: 0° or 17° for anterior implants and 30° for posterior tilted implants. Both digital and analog impressions were analyzed, with the operator having the flexibility to select either method according to his preference. All the analog impressions and intraoral scans were taken by the same clinician with more than 5 years of experience in full‐arch immediate loading rehabilitation.

Flaps were closed using resorbable sutures (4/0 Vicryl Ethicon, Somerville, NJ, USA). For patients undergoing digital impressions, scan abutments (Straumann CrossFit) were connected to the multi‐unit abutments, and a full‐arch digital impression was acquired using the Trios 3 Shape scanner (København K, Capital Region, Denmark). In cases where analog impressions were performed, pick‐up transfers were secured onto the abutment platforms and stabilized using interdental floss as a framework for cyanoacrylate fixation (Periacryl High Viscosity GluStich, Canada) or flowable light‐curing composite (Bulkfill Kyoto, Japan). Impressions were taken using standard plastic open trays and polyvinyl siloxane material (Flexitime Regular and Heavy body Kulzer, Hanau, Germany).

The time employed to take the impressions (analog or digital) was recorded with a chronometer starting from the connection of the pick‐up transfers for the analog technique or from the connection of the scanbodies in the case of the digital one. The end was considered once the impression tray was removed from the patient's mouth or the digital file was correctly delivered to the dental laboratory.

Once impressions were obtained, any remaining teeth were extracted, and patients were discharged with a scheduled appointment for the temporary bridge delivery within 24 h post‐surgery. The screw‐retained full‐arch bridge was fabricated from printed acrylic resin and reinforced with a titanium‐milled bar, without cantilevered extensions.

**FIGURE 3:**
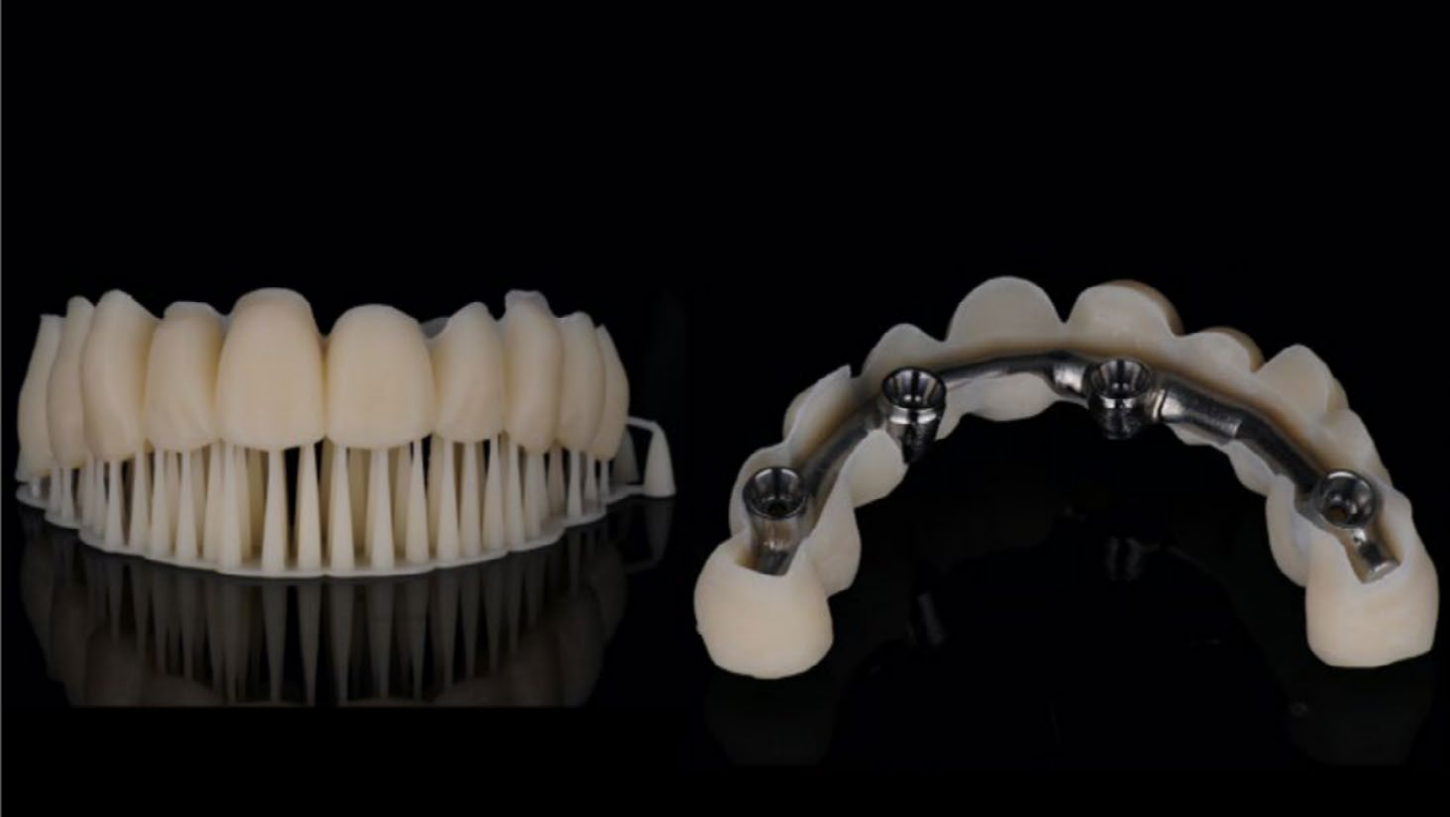
PMMA structure and titanium bar inside the PMMA.

Patients were monitored in follow‐up visits, and the final prosthetic restoration was delivered 130 days after implant placement using the same impression technique selected at the beginning. All the final restorations were made of milled titanium bar embedded in a PMMA printed supra‐structure, which was designed and produced with a full digital workflow (Figure 3).

**FIGURE 4:**
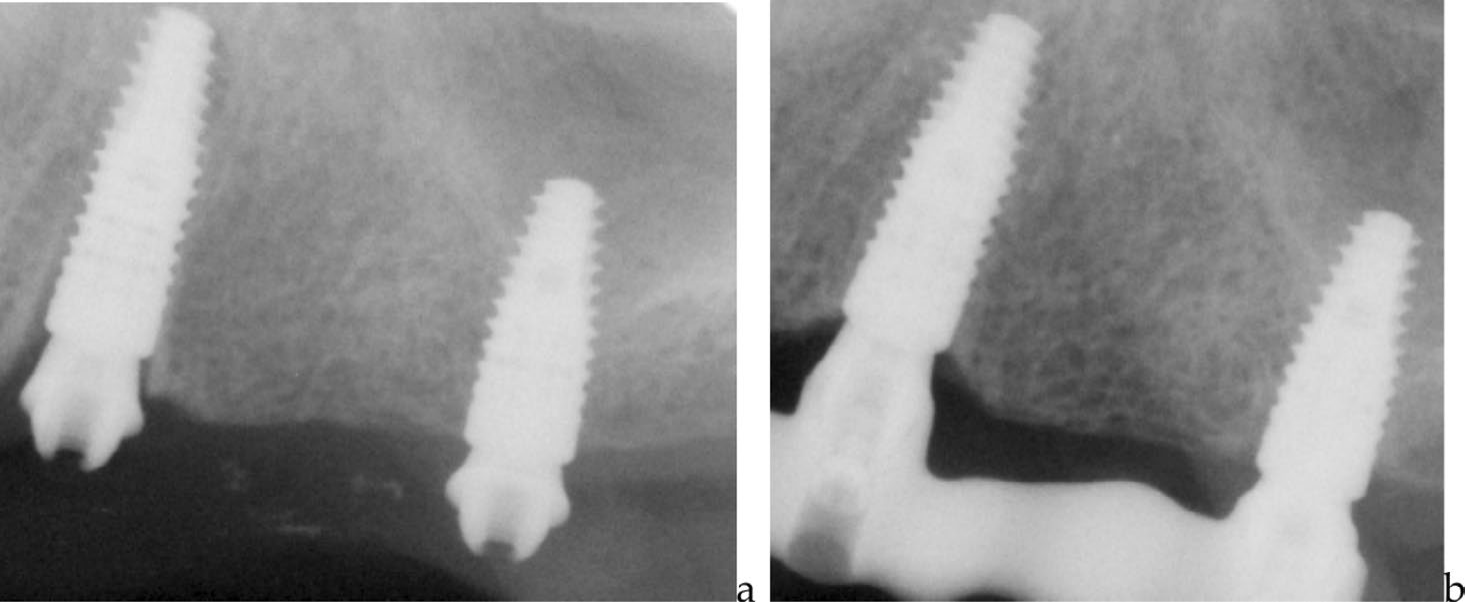
(a) T0 peri‐apical X‐ray of one case (immediately after implant placement); (b) the same case at T3 (5 years post loading).

Additional check‐ups were performed at 2‐ and 5‐year post‐loading, during which digital periapical radiographic examinations were repeated using the long‐cone parallel technique (Figure 4).

Bone level was assessed at specific time points: T0 (immediately post‐implant placement), T1 (after loading—130 days that is at the delivery of the definitive prosthesis), T2 (2 years post‐loading), and T3 (5 years post‐loading) measuring the distance between the implant shoulder and the most coronal bone‐implant contact on the mesial and distal side of each implant using the software Image J. Measurements were based on the known distance from the implant shoulder to the first thread and the spacing between threads, as provided by the implant manufacturer. At each time point, also, Full Mouth Plaque Score (FMPS) was recorded [30].

Any biologic or technical complication was recorded.

### 2.3 Statistical Analysis

Statistical analysis included repeated measure ANOVA (RMA) to investigate changes in bone level at T0, T1, T2, and T3, representing respectively the marginal bone level values at implant placement, after loading (final prosthetic treatment), and at 2‐ and 5‐years of follow‐up, to support full‐arch prosthetists. As an assumption, sphericity was evaluated and tested by Mauchly's test of Sphericity. In case of violation, *p*‐value < 0.05, corrections were applied, such as Greenhouse–Geisser correction *ε* for values below 0.60 and Huynh‐Feldt one for values equal to or above 0.60 as suggested by Blanca et al. [31].

Normality was tested by the Shapiro–Wilk test. As most of the primary variables fell into a normal distribution, RMA was employed to investigate the differences in marginal bone loss values among different time points. Multiple comparisons *p*‐values were evaluated by the Bonferroni post hoc test. RMA was also employed in the case of non‐normal distribution as it is demonstrated that relative type I error is not altered by the violation of normality [32]. Differences in baseline characteristics were also investigated by the Chi‐square test for dichotomous variables, while the *t*‐test for independent samples was used to investigate also the differences of age and FMPS at baseline between the treatment groups (analog vs. digital). Lastly, the time for taking the impression was recorded, and differences between the analog and digital workflows were recorded and analyzed.

## 3 Results

Distribution of baseline variables between the two groups is displayed in Table 1.

**TABLE 1:** Clinical characteristics of patients at baseline distributed between the analog and digital workflow.

	Analog				Digital				*p*
	Count	*N* %	Mean	Standard deviation	Count	*N* %	Mean	Standard deviation	
Sex									
Female	11	35.5%			13	41.9%			0.602
Male	20	64.5%			18	58.1%			
Jaw									
Lower	20	64.5%			18	58.1%			0.602
Upper	11	35.5%			13	41.9%			
Opposite_jaw									
Fixed full‐arch	12	38.7%			10	32.3%			0.84
Natural dentition	11	35.5%			13	41.9%			
Removal denture	8	25.8%			8	25.8%			
Outcome									
Success	26	83.9%			26	83.9%			1
Failure	5	16.1%			5	16.1%			
Age			69.38	6.9			68.64	5.8	0.648
Full mouth plaque score (%)			17	4			21	5	0.005
Mean_bone_level_t0			0.21	0.04			0.17	0.04	< 0.001
Time_impression (min)			51	3			31	4	< 0.001
Additional_time (min)			44	5			29	5	< 0.001

Records of a total of 62 patients, who underwent full‐arch rehabilitation with simultaneous extractions and immediate dental implant placement without restrictions of position and site (upper and lower jaw) between January 2019 and January 2020, were included in the study.

At baseline, no differences were found between the investigated groups and sex (Chi‐square *p*‐value = 0.602), upper or lower jaw (Chi‐square *p*‐value = 0.602), and type of opposite dentition (such as, fixed full‐arch, removable denture or natural dentition, Chi‐square *p*‐value = 0.840). Mean age did not differ (*t*‐test *p*‐value = 0.648). FMPS at baseline differed between groups and was higher in the digital workflow group (mean = 20.65, SD = 4.8 vs. analog mean = 17.39, SD = 4.0, *t*‐test *p*‐value = 0.005) as well as the marginal bone level at t0 after implant placement, which was higher in the analog group (mean = 0.21, SD = 0.04 vs. digital mean = 0.17, SD = 0.04, *t*‐test *p*‐value < 0.001). At 5 years follow‐up, no differences were found in terms of failures with five events (veneering material fracture, screw loosening, internal screw fracture, bar fracture, implant fracture) respectively found in each group (Chi‐square test *p*‐value = 1.0). Overall, the analog workflow required longer time, even for the additional procedures (such as repeating the impression, fixing again the transfers [*t*‐test *p*‐value < 0.001]). Of interest, when looking at which employed workflow required additional procedures, no statistically significant differences were found, as these were required in respectively 10 cases for each group (Chi‐square test *p*‐value = 1.0).

**FIGURE 5:**
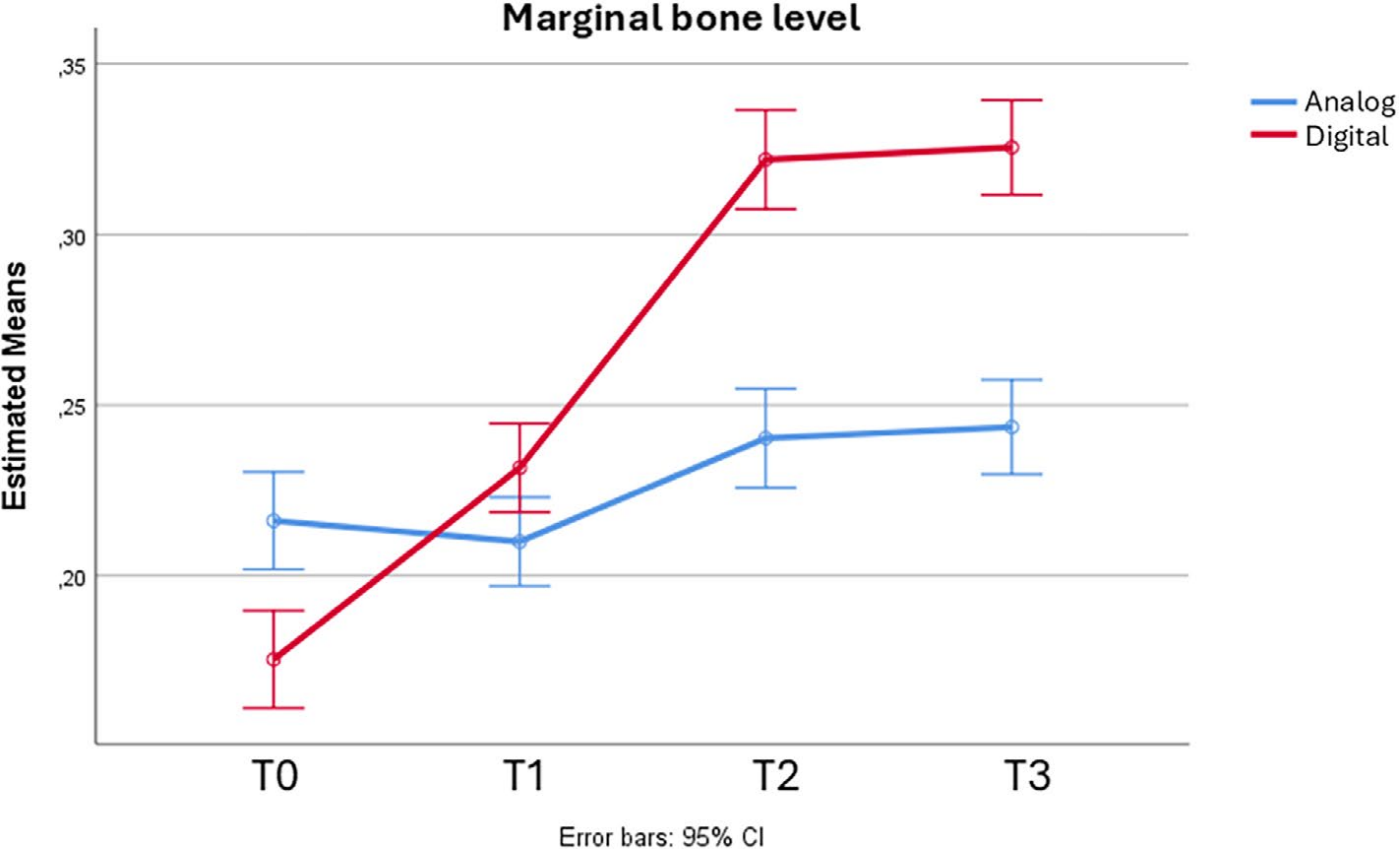
Graphical representation of MBL at different time intervals.

Overall MBL increased over time in both groups. In the analog impression group, the mean value at T0 was 0.21 mm, SD = 0.04, similar to T1, 0.21 mm, SD = 0.04, and increased at T2 and T3, respectively 0.24 mm, SD = 0.02, and 0.24 mm, SD = 0.02. Despite the recorded MBL in the digital group (0.17 mm, SD = 0.04) being inferior to the analog one at T0, a higher increase in this value was observed over time, and values at T1, T2, and T3 were respectively 0.23 mm, SD = 0.03, 0.32 mm, SD = 0.05, and 0.32 mm, SD = 0.05 (Table 2). As Mauchly's *p*‐value was < 0.001, sphericity was not assumed, so corrections were applied. The RMA corrected with Huynh‐Feldt test (*ε* = 0.629) showed a statistically significant increase over time of MBL (*p*‐value < 0.001); moreover, this effect was impacted by the treatment group (*p*‐value < 0.001) (Figure 5).

**TABLE 2:** Mean marginal bone level at T0, T1, T2, and T3 per group treatment and total.

	Digital_analog	Mean	Std. deviation
Mean_bone_level_T0	Analog	0.2161	0.03997
	Digital	0.1755	0.03948
	Total	0.1958	0.04441
Mean_bone_level_T1	Analog	0.2100	0.04131
	Digital	0.2316	0.03001
	Total	0.2208	0.03743
Mean_bone_level_T2	Analog	0.2403	0.02562
	Digital	0.3219	0.05115
	Total	0.2811	0.05746
Mean_bone_level_T3	Analog	0.2435	0.02537
	Digital	0.3255	0.04829
	Total	0.2845	0.05630

Bonferroni correction for multiple comparison confirmed these differences in MBL. While in the analog group there were no differences between T0 and T1, in the digital group there was a statistical increase in MBL already at T1, with a statistical increase in this group at all time points but in the comparison T3 versus T2. In the analog group a different trend was noticed with a statistically significant increase in the comparisons of T2 and T3 versus T0 and T1, but not when comparing T2 and T3 (Table 3).

**TABLE 3:** Multiple pairwise comparisons between the two treatments among the different time points (*p*‐value adjusted per Bonferroni correction).

Pairwise comparisons							
Measure	Mean_bone_level						
Digital_analog			Mean difference (I − J)	Std. error	Sig.^a^	95% confidence interval for difference^a^	
						Lower bound	Upper bound
Analog	T0	T1	0.006	0.007	1.000	−0.014	0.026
		T2	−0.024	0.009	0.052	−0.048	0.000
		T3	−0.027^b^	0.009	0.014	−0.051	−0.004
	T1	T0	−0.006	0.007	1.000	−0.026	0.014
		T2	−0.030^b^	0.007	0.001	−0.051	−0.010
		T3	−0.034^b^	0.007	0.000	−0.054	−0.014
	T2	T0	0.024	0.009	0.052	0.000	0.048
		T1	0.030^b^	0.007	0.001	0.010	0.051
		T3	−0.003	0.002	0.266	−0.008	0.001
	T3	T0	0.027^b^	0.009	0.014	0.004	0.051
		T1	0.034^b^	0.007	0.000	0.014	0.054
		T2	0.003	0.002	0.266	−0.001	0.008
Digital	T0	T1	−0.056^b^	0.007	0.000	−0.076	−0.036
		T2	−0.146^b^	0.009	0.000	−0.171	−0.122
		T3	−0.150^b^	0.009	0.000	−0.173	−0.127
	T1	T0	0.056^b^	0.007	0.000	0.036	0.076
		T2	−0.090^b^	0.007	0.000	−0.111	−0.070
		T3	−0.094^b^	0.007	0.000	−0.114	−0.074
	T2	T0	0.146^b^	0.009	0.000	0.122	0.171
		T1	0.090^b^	0.007	0.000	0.070	0.111
		T3	−0.004	0.002	0.165	−0.008	0.001
	T3	T0	0.150^b^	0.009	0.000	0.127	0.173
		T1	0.094^b^	0.007	0.000	0.074	0.114
		T2	0.004	0.002	0.165	−0.001	0.008

*Note:* Based on estimated marginal means.

^a^ Adjustment for multiple comparisons: Bonferroni.

^b^ The mean difference is significant at the 0.05 level.

## 4 Discussion

The present retrospective clinical study suggests that implant‐supported full‐arch restorations represent a viable option for full‐arch rehabilitation, which complies with other evidence recently published [33].

All the prostheses (both temporary and final) analyzed in the study were made of acrylic resin with a milled titanium bar inserted and cemented inside the structure. There is still a high debate on which prosthesis material can rely on the best clinical outcomes over time in full‐arch rehabilitations. A recently published systematic review [34] reported that different materials can be used, but some of them, such as zirconia, might present some issues and their use for implant‐supported full‐arch restorations is not yet well documented in the long‐term follow‐up, which is also in agreement with other previously published papers [2].

In a recent review [35, 36] the authors report that analog impression techniques are still recommended for full‐arch rehabilitations. Interestingly, another clinical study published in 2023 by the same authors of the present manuscript [13] compares the outcomes for analog and digital impressions in terms of marginal gap evaluation on the abutment‐bar junction, and the results reported a statistically significant lower gap for the digital group (analyzed patients were also part of this study). Similar findings were also corroborated by a further systematic review [37]. However, in the previously published paper [12] at the 2‐year follow‐up, marginal bone level significantly differed between patients rehabilitated using an intraoral scan and those rehabilitated using an analog impression. This is in contrast with the present outcomes. Reasons may be attributed to the longer follow‐up period; indeed, the statistical increase in the digital group was noticed in the T2 versus T3 comparison.

Another possible reason might be the marginal gap which was not measured in this study, therefore it is not possible to determine if differences in the clinical outcomes might be related to differences in prostheses fit. It is important to acknowledge that detecting marginal bone changes as small as 0.03 mm is methodologically challenging, and such minimal variations may fall within the limits of measurement error inherent to current radiographic techniques. Therefore, these findings should be interpreted with caution and within the context of the resolution capabilities of the imaging methods used.

In the present retrospective study, the authors aimed to quantify also the time spent for the impression taking, reporting also the additional procedures (such as repeating the impression when not satisfactory). The results revealed that for digital impressions the time required is almost half of the one needed for analog impressions (31 vs. 51 min) and in case of additional procedures the analog impression exceeds by 44 versus the 29 min of the digital group. Similar results were also reported in this systematic review [38].

Although the analog group exhibited a lower bone loss at T2 and T3 compared to the digital group, it must be underlined that both groups showed a minimal bone loss: 0.32 and 0.24 mm for the digital and analog groups, respectively, which is within normal limits. The authors consider this difference not significant from the clinical point of view.

Among the limitations of the present research, it must be underlined that its retrospective design and therefore the assignment to the two groups (analog vs. digital) has not been randomized. This might have introduced bias possibly affecting the outcomes since several other factors, besides the impression technique, might affect peri‐implant bone level, including patients' characteristics, such as patient general health, oral hygiene habits, characteristics of the bone site, etc. However, it must be underlined that the two groups did not differ in sex, jaw treated (upper or lower), type of antagonist arch, and mean age at T0. In contrast, FMPS at T0 significantly differed between the two groups. This is remarkable since, although this is not universally accepted [39], plaque accumulation is generally considered one of the principal risk factors for peri‐implant bone loss and peri‐implantitis.

On the other side, among the merits of the investigation it must be underlined that all the clinical procedures were conducted by the same clinician with more than 5 years of experience in full‐arch immediate loading rehabilitation; therefore, possible variables related to the clinician's learning curve were excluded. In addition, always the same implant type was inserted, so that variables related to implant macro‐ and micro‐design, implant connection, surface characteristics, etc. were eliminated [40]. As future trends, research should aim to conduct RCTs with different treatment modalities, digital workflows, and guided surgery or dynamic navigation.

This retrospective clinical study confirms that implant‐supported restorations are a viable option for full‐arch rehabilitation, aligning with existing literature. Analog impression techniques continue to be recommended in this kind of rehabilitation and showed optimal outcomes with lower bone loss at the 5‐year follow‐up compared to intraoral scanning in the present study, although both groups remained within clinically acceptable limits. On the other hand, intraoral scanning demonstrated advantages, particularly improving procedural efficiency. In fact, it required significantly less time than analog methods, particularly when additional procedures were needed.

## 5 Conclusions

Analog impression techniques continue to be recommended in this kind of rehabilitation and show optimal outcomes with lower bone loss at the 5‐year follow‐up compared to intraoral scanning, while digital impressions offer efficiency benefits, lower working time, and potential clinical advantages. Future randomized, long‐term studies are needed to further evaluate the impact of impression techniques on treatment outcomes in the case of full‐arch immediate loading rehabilitations.

## Conflicts of Interest

The authors declare no conflicts of interest.

## Supporting information


**Data S1.** STROBE checklist.

## Data Availability

The data that support the findings of this study are available on request from the corresponding author. The data are not publicly available due to privacy or ethical restrictions.
